# Supplementary material for: High resolution long-read telomere sequencing reveals dynamic mechanisms in aging and cancer
Source: Nat Commun. 2024 Jun 18;15:5149. doi: 10.1038/s41467-024-48917-7 (PMC11189484; doi:10.1038/s41467-024-48917-7)
Supplement: Supplementary file 1 — Supplementary Information [file 41467_2024_48917_MOESM1_ESM.pdf]

Supplementary Information for

High resolution long-read telomere sequencing reveals dynamic mechanisms in aging and cancer

Tobias T. Schmidt, Carly Tyer, Preeyesh Rughani, Candy Haggblom, Jeffrey R. Jones, Xiaoguang Dai, Kelly A. Frazer, Fred H. Gage, Sissel Juul, Scott Hickey*, and Jan Karlseder*

*Corresponding should be addressed to Jan Karlseder (karlseder@salk.edu) or Scott Hickey (scott.hickey@nanoporetech.com)

This file includes:

Supplementary Figures 1 to 9
Supplementary Tables 1 to 7

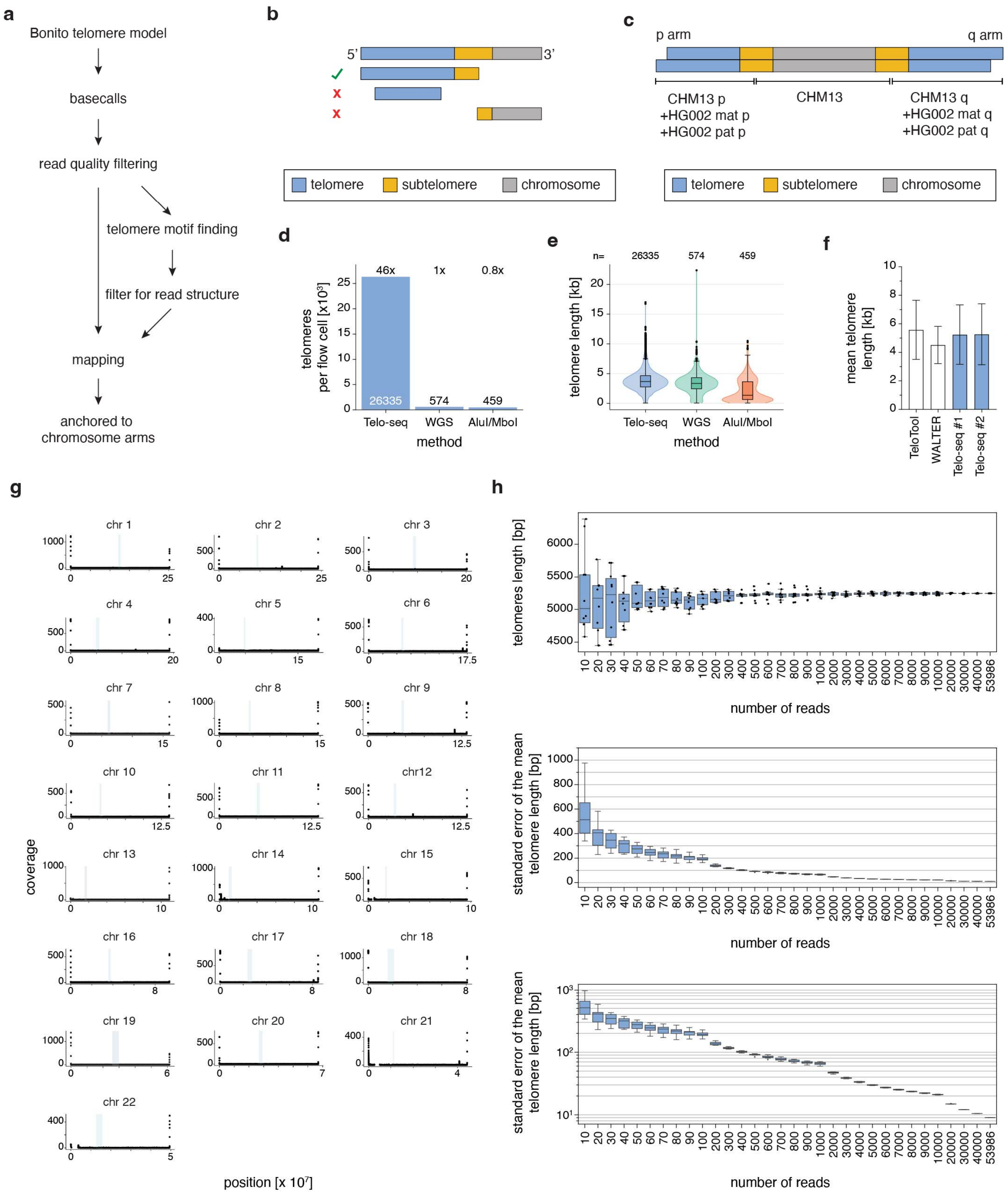

Supplementary Fig. 1. Telo-seq analysis exemplified on HG002. a, Scheme of bioinformatic Telo-seq reads analysis. **b,** Scheme of the read structure filter. Only reads with a terminal telomeric sequence and an adjacent unique, non-telomeric sequence pass the read structure filter. **c,** Scheme of combined assembly used to anchor telomeric reads to specific chromosome arms. CHM13 and HG002 were used for each chromosome arm. **d,** Bar graph comparing the number of HG002 telomeric reads per flow cell with either Telo-seq, whole genome sequencing

(WGS) or AluI/MboI restriction enzyme libraries. Fold increase over WGS is given above bar. Libraries were prepared from the same HG002 cell pellet. **e**, Violin plots of bulk HG002 telomere length measurement by Telo-seq, WGS and AluI/MboI libraries as shown in Supplementary Fig. 1d. Boxplot shows the median telomere length with interquartile range (IQR) and whiskers represent 1.5-fold IQR. The number of telomeric reads per sample is shown above the plot. The HG002 passage was different than in Fig. 1 and Supplementary Fig. 1f-h and 2. **f,** Bar graph of HG002 mean telomere length with standard deviation in kilobase (kb). Telomere length comparison of TeloTool and WALTER telomere length quantification of Terminal Restriction Fragment analysis (Fig. 1b) and Telo-seq results of both HG002 replicates (Supplementary Table 1). **g**, Binned coverage plots per chromosome of telomeric and non-telomeric reads obtained by both HG002 Telo-seq runs. The rolling median is plotted with a bin size of 2.5 kb. Centromeres are shown in light blue. **h**, Simulated telomere length based on HG002 Telo-seq telomere length distribution. For each condition, the indicated number of reads are drawn from HG002 Telo-seq data telomeric read distribution and each condition is performed 10 independent times. Top graph, the boxplot shows simulated median HG002 telomere length with IQR and whiskers represent 1.5-fold IQR for the indicated number of reads. Dots represent individual simulations. Middle graph, the boxplot shows the median standard error of the mean with IQR and whiskers represent 1.5-fold IQR for the indicated number of reads. Bottom graph, as middle graph, but y-axis is log10-transformed. d, e, h: one experiment; f, g: two experiments.

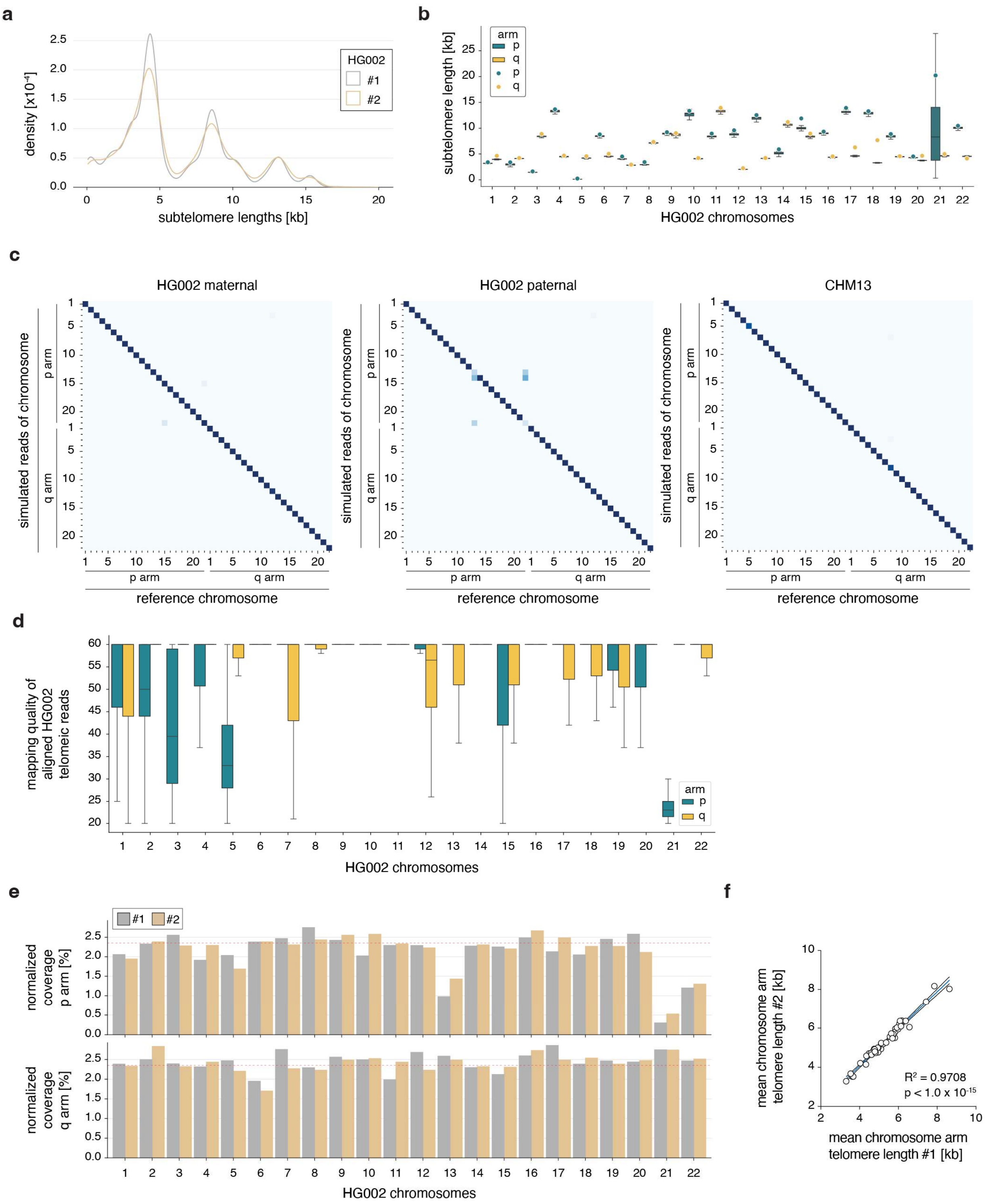

Supplementary Fig. 2. Telo-seq chromosome arm-specific analysis exemplified on HG002. **a,** Subtelomeric length density for the two independent replicates of HG002. Subtelomeric length is given in kilobase (kb). **b**, Boxplot of HG002 Telo-seq subtelomere length per chromosome arm. Middle line represents median, the box the interquartile range (IQR) and whiskers the 1.5-fold

IQR. The calculated subtelomere length based on the EcoRV in silico digested HG002 reference genome is shown as circles. **c**, Matrix heatmap of simulated HG002 and CHM13 telomeric reads mapping to their respective reference genome. Per chromosome arm, 30 independently simulated reads were used with varying telomere length and a 2% error rate. **d**, Boxplot of chromosome arm-specific mapping quality for HG002 Telo-seq reads. The middle line represents median, the box the IQR and whiskers represent 1.5-fold IQR. **e**, Bar graph of the normalized coverage of mapped telomeric reads per chromosome arm for both HG002 replicates in percent. Mapped Q20+ telomeric reads per chromosome arm are normalized to total mapped Q20+ telomeric reads. The median coverage for all chromosome arms is shown as red dotted line. **f,** Scatter plot of mean chromosome arm-specific telomere length of HG002 replicate 2 against replicate 1. Linear regression analysis was performed. Blue line represents best fit and black curves 95% confidence intervals. a, b, d-f: two experiments; c: one experiment.

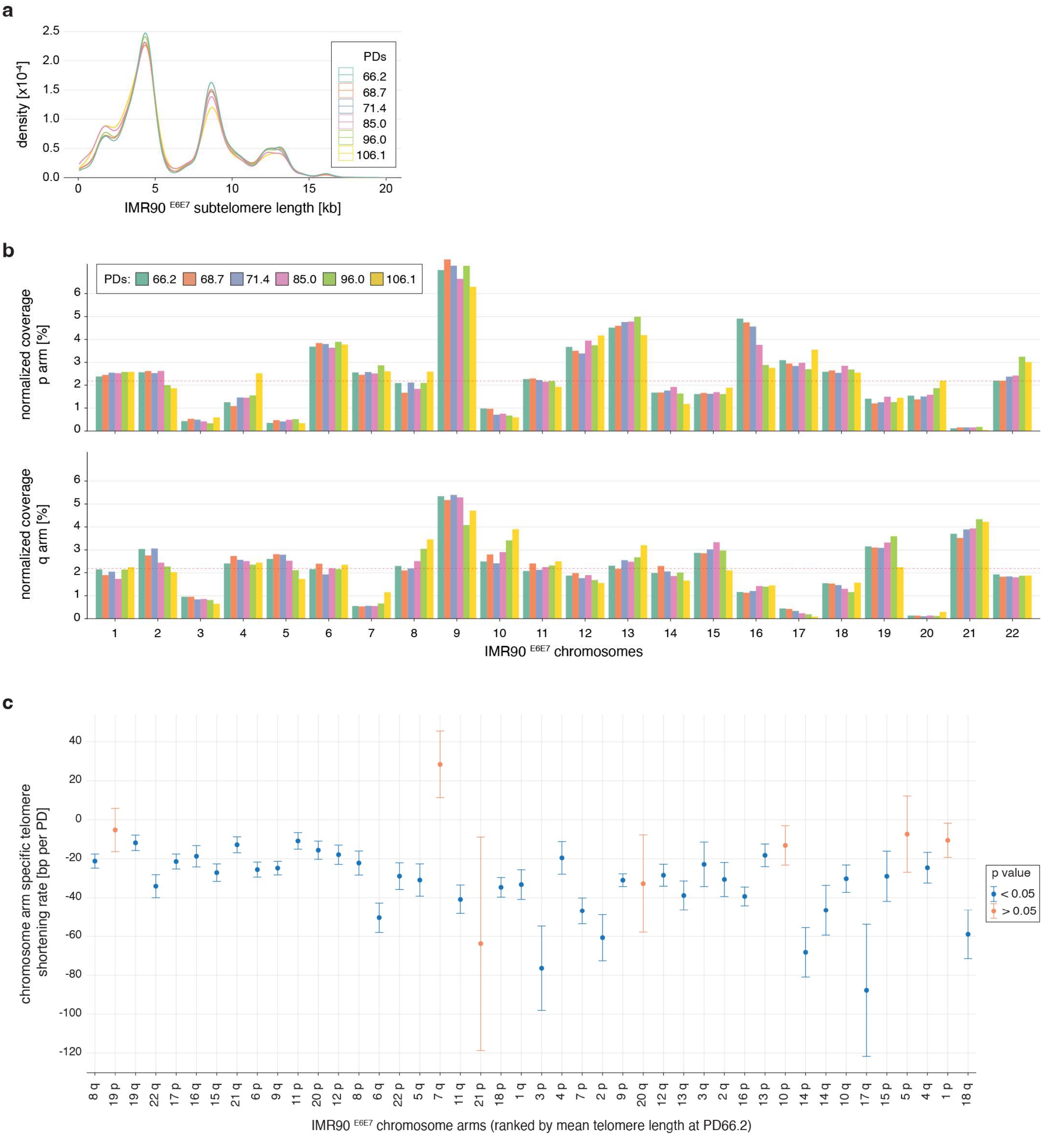

Supplementary Fig. 3. Telo-seq analysis of telomere shortening in IMR90 ^{E6E7} fibroblasts. **a,** Subtelomeric length density of IMR90 ^{E6E7} fibroblasts at different population doublings (PD). Subtelomeric length is given in kilobase (kb). **b,** Bar graph of the normalized coverage of mapped telomeric reads per chromosome arm for IMR90 ^{E6E7} at different PDs in percent. Mapped Q20+ telomeric reads per chromosome arm are normalized to total mapped Q20+ telomeric reads. The median coverage for all chromosome arms is shown as red dotted line. **c,** Chromosome arm-specific telomere shortening rate in base pairs (bp) per PD based on linear regression analysis of chromosome arm-specific telomere length of IMR90 ^{E6E7} from PD66.2 to PD85.0. Telomere

shortening rate per PD is shown with standard error. Chromosome arms are ranked based on mean telomere length in IMR90 ^{E6E7} at PD66.2. a-c: one experiment.

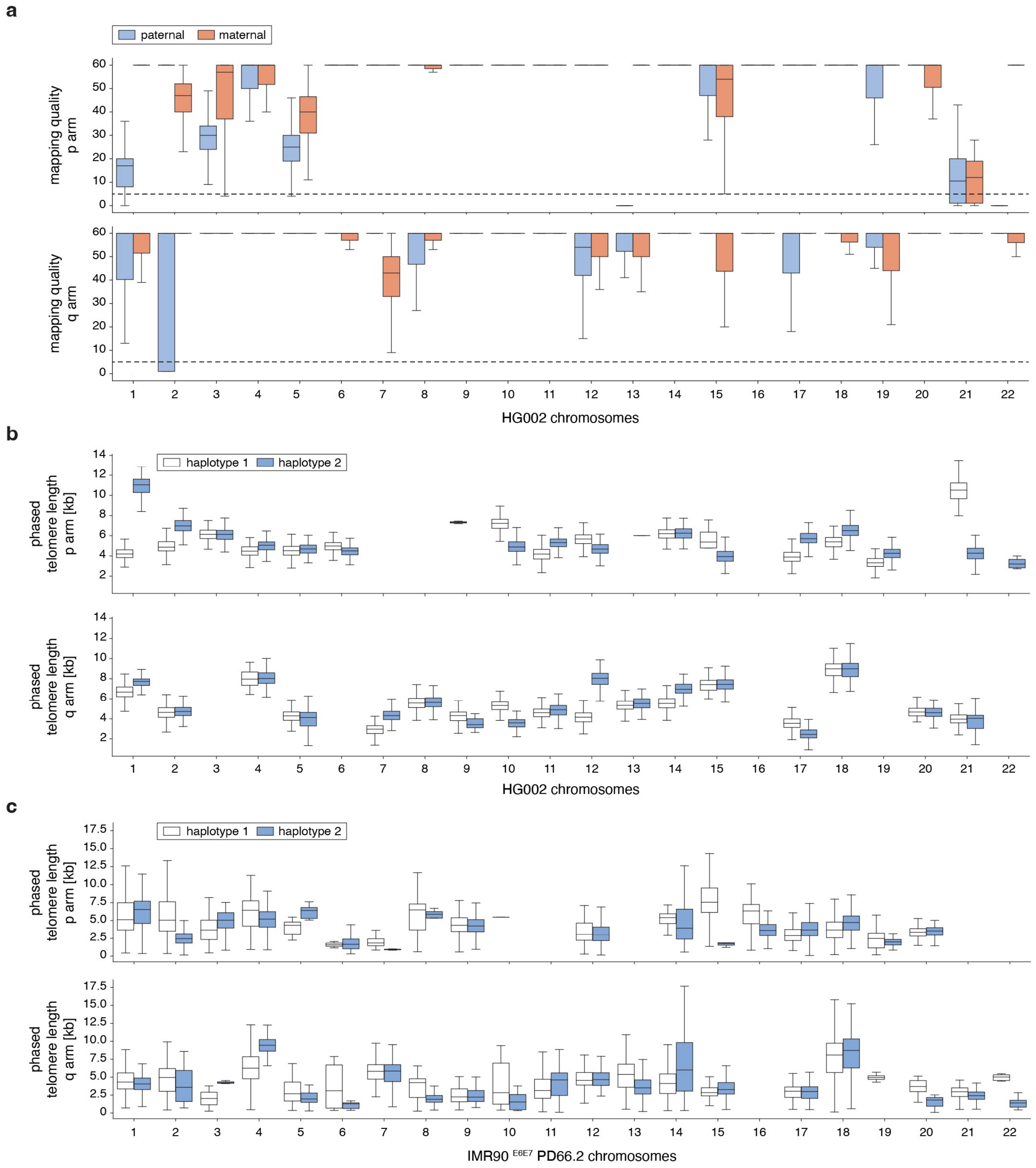

Supplementary Fig. 4. Allele specific telomere length of HG002 and IMR90 ^{E6E7}. a, Boxplot of mapping quality of HG002 allele-specific telomere length (Fig. 3). The middle line represents the median, the box the interquartile range (IQR) and the whiskers the 1.5-fold IQR. The horizontal dashed line represents the mapping quality threshold used in Fig. 3. **b,** Boxplot of allele-specific telomere length in kilobase (kb) of HG002 based on de-novo haplotype phasing. The middle line represents median, box the IQR and whiskers the 1.5-fold IQR. **c,** Boxplot of allele-specific telomere length in kb of IMR90 ^{E6E7} at population doubling (PD) 66.2 based on de-novo haplotype phasing. The middle line represents median, box the IQR and whiskers the 1.5-fold IQR. For a

and b, only the alleles that passed the filter were included in the figure, see methods for details. a, b: based on two experiments; c: one experiment.

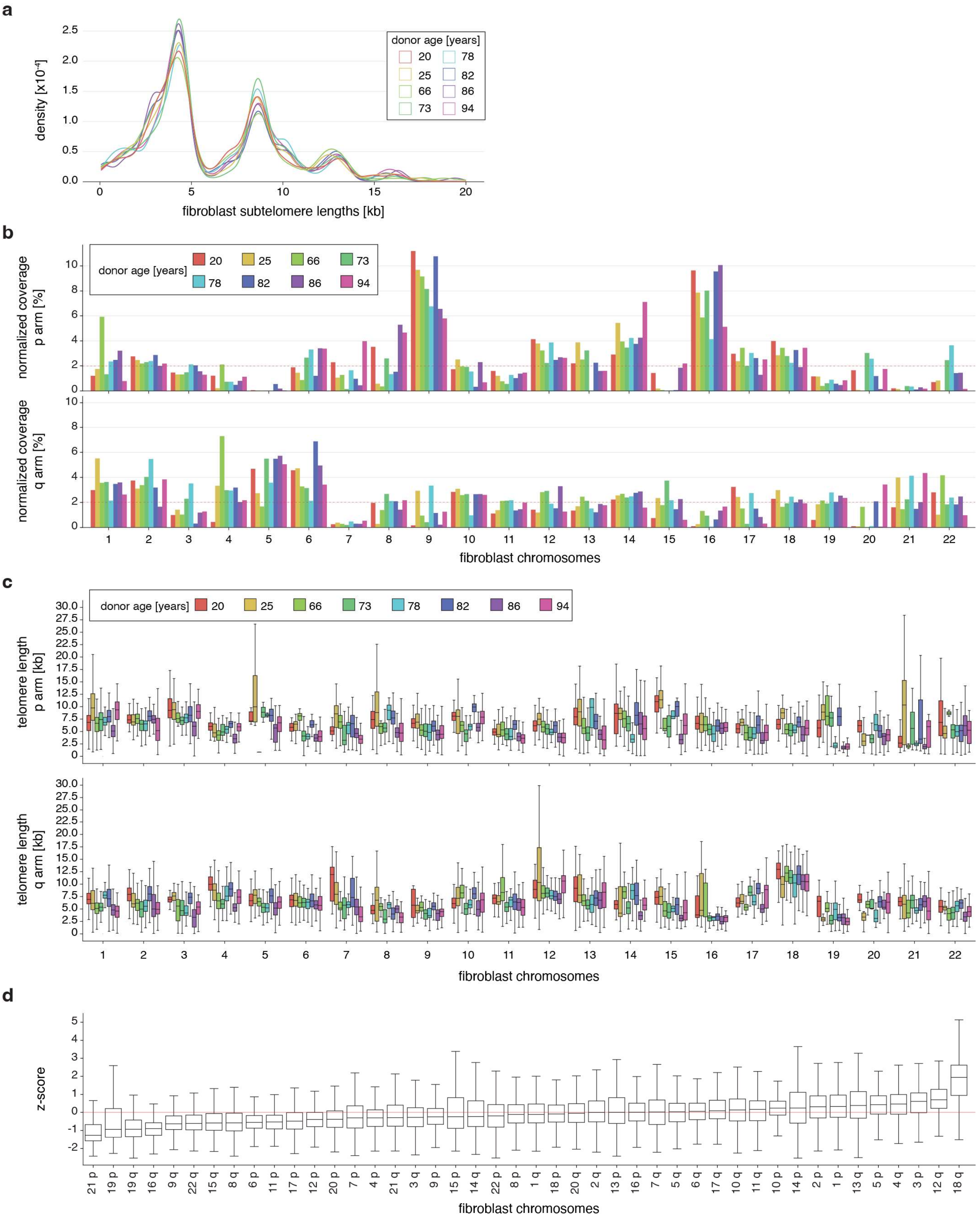

Supplementary Fig. 5. Telo-seq on aging cohort fibroblasts. a, Subtelomeric length density of donor-derived fibroblasts of the indicated age. Subtelomeric length is given in kilobase (kb). **b,** Bar graph of the normalized coverage of mapped telomeric reads per chromosome arm of donor-

derived fibroblasts of the indicated age in percent. Mapped Q20+ telomeric reads per chromosome arm are normalized to total mapped Q20+ telomeric reads. The median coverage for all chromosome arms is shown as red dotted line. **c,** Boxplot of chromosome arm-specific telomere length of donor-derived fibroblasts with the indicated donor age. The middle line represents median telomere length, box the interquartile range (IQR) and whiskers the 1.5-fold IQR. For limitations on Telo-seq chromosome arm-specific telomere length assignment see "Discussion". **d,** z-score analysis of fibroblast chromosome arm-specific telomere length of donor-derived fibroblasts and IMR90 ^{E6E7} PD66.2. The middle line represents median z-score, box the IQR and whiskers the 1.5-fold IQR. Chromosome arms are ranked according to their median z-score. The z-score of the median bulk telomere length of all nine fibroblast samples is shown as red line. See Supplementary Data 2 and 4. a-d: one experiment.

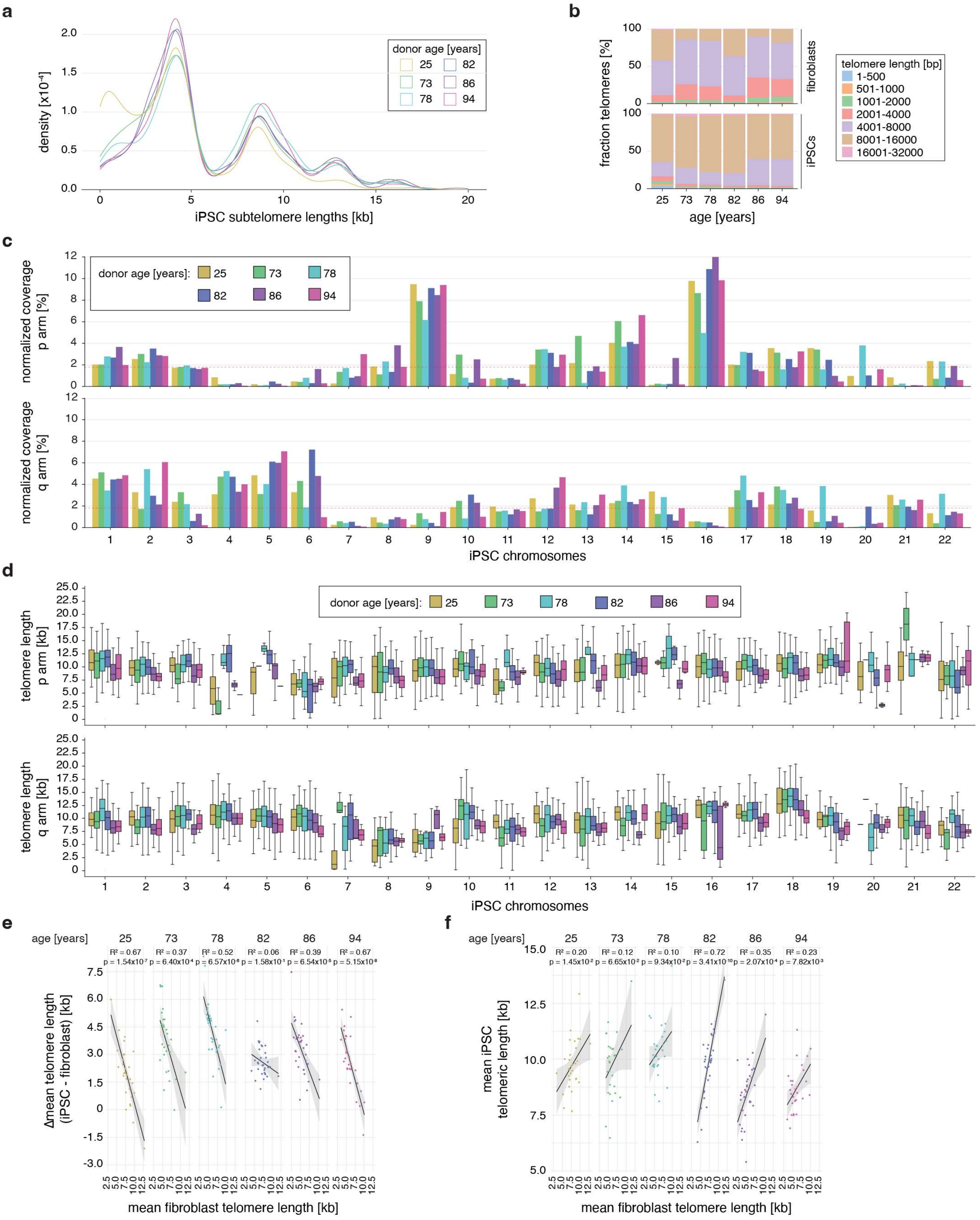

Supplementary Fig. 6. Telo-seq on matched iPSCs. a, Subtelomeric length density of matched induced pluripotent stem cells (iPSC). Subtelomeric length is given in kilobase (kb). **b,** Bar graph showing the percentage of binned telomere length in donor-derived matched fibroblasts (top) and

iPSC (bottom). **c,** Bar graph of the normalized coverage of mapped telomeric reads per chromosome arm of matched iPSCs of the indicated donor age in percent. Mapped Q20+ telomeric reads per chromosome arm are normalized to total mapped Q20+ telomeric reads. The median coverage for all chromosome arms is shown as red dotted line. **d,** Boxplot of chromosome arm-specific telomere length in kb of matched iPSCs with the indicated donor age. The middle line represents median, box the interquartile range (IQR) and whiskers the 1.5-fold IQR. For limitations on Telo-seq chromosome arm-specific telomere length assignment see “Discussion”. **e,** Linear regression analysis of the difference between the mean chromosome arm-specific telomere length of matched iPSC and fibroblasts against the mean chromosome arm-specific telomere length of fibroblasts in kb. Black line represents best fit and grey area the 95% confidence interval. Only chromosome arms with at least 20 reads per sample were included. **f,** Linear regression analysis of the mean chromosome arm-specific telomere length of matched iPSC against fibroblasts in kb. Black line represents best fit and grey area the 95% confidence interval. Only chromosome arms with at least 20 reads per sample were included. See Supplementary Data 4 and 5. a-f: one experiment.

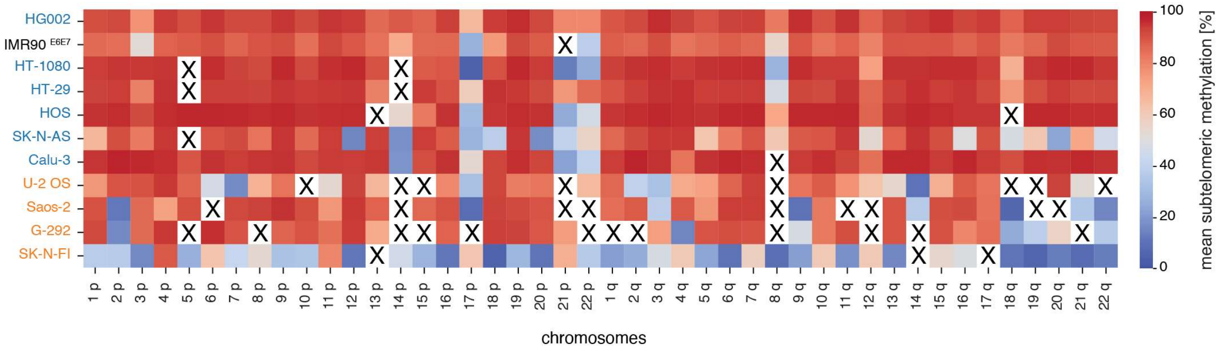

Supplementary Fig. 7. Subtelomeric methylation. Heatmap of the mean subtelomeric CpG site methylation of the indicated chromosome arm and cell lines. For IMR90 ^{E6E7} the methylation at population doubling 66.2 is shown. Mean methylation is shown for the chromosome arms that have at least 10 reads with 100 CpG sites in the read. Chromosome arms that do not fulfill that criteria are labeled with a black “X” on white background. Data is based on the pooled analysis of one or two experiments per cell line. See Supplementary Tables 1, 2 and 6 for details.

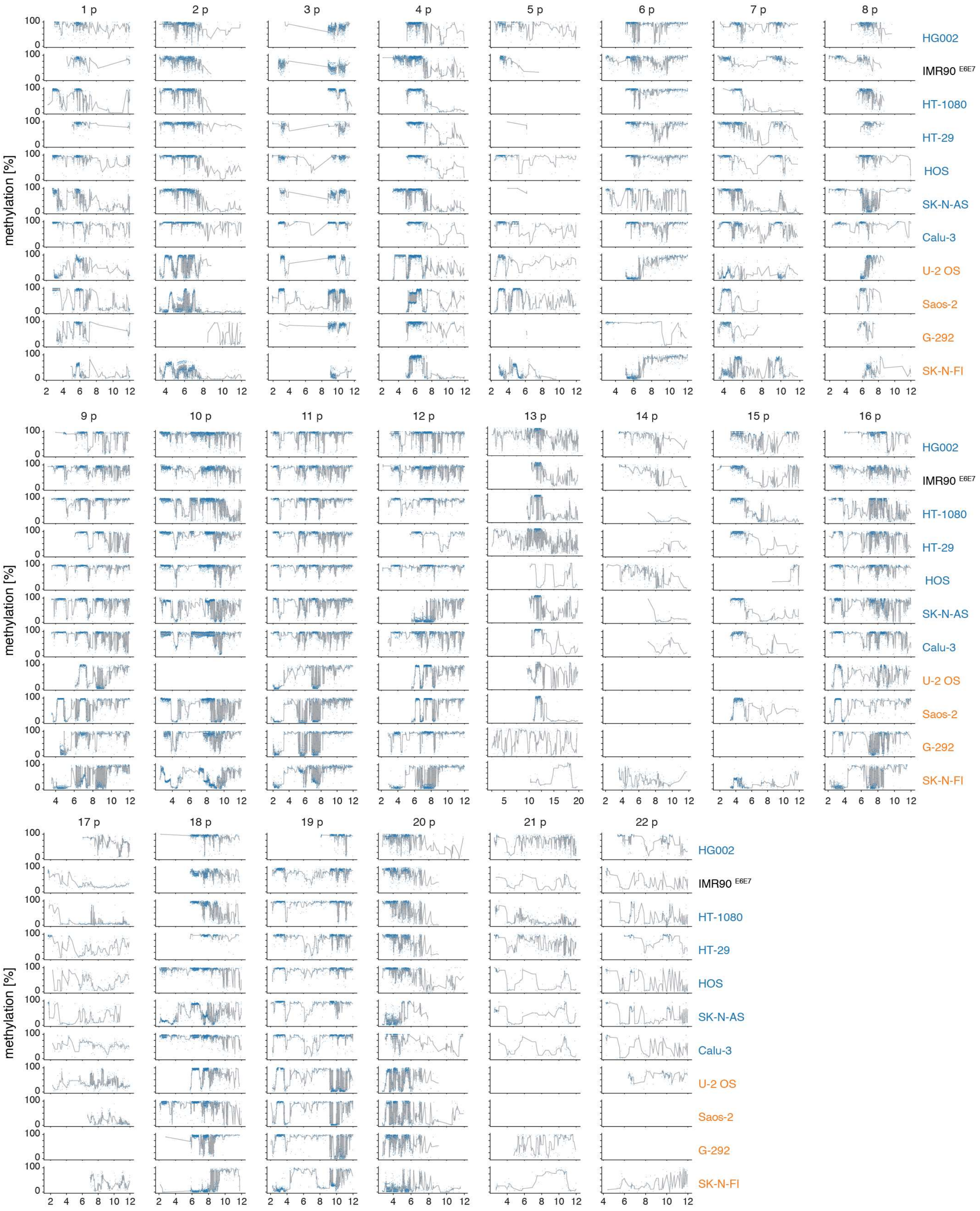

Supplementary Fig. 8. Subtelomeric methylation p chromosome arms. Line graphs of subtelomeric CpG islands closest to telomeres. The percentage of methylation is shown for indicated cell lines and chromosome arms at their kilobase position. Individual CpG sites are represented by blue points and the rolling methylation median is shown as a gray line with a

window size of 4. For IMR90 ^{E6E7} the methylation at population doubling 66.2 is shown. Cell lines are color-coded according to telomere maintenance mechanism (TMM); cells with no TMM are in black, telomerase-positive cells in blue and ALT-positive cells in orange. Only data for chromosomes with at least 10 reads per arm are shown. Data is based on the pooled analysis of one or two experiments per cell line. See Supplementary Tables 1, 2 and 6 for details.

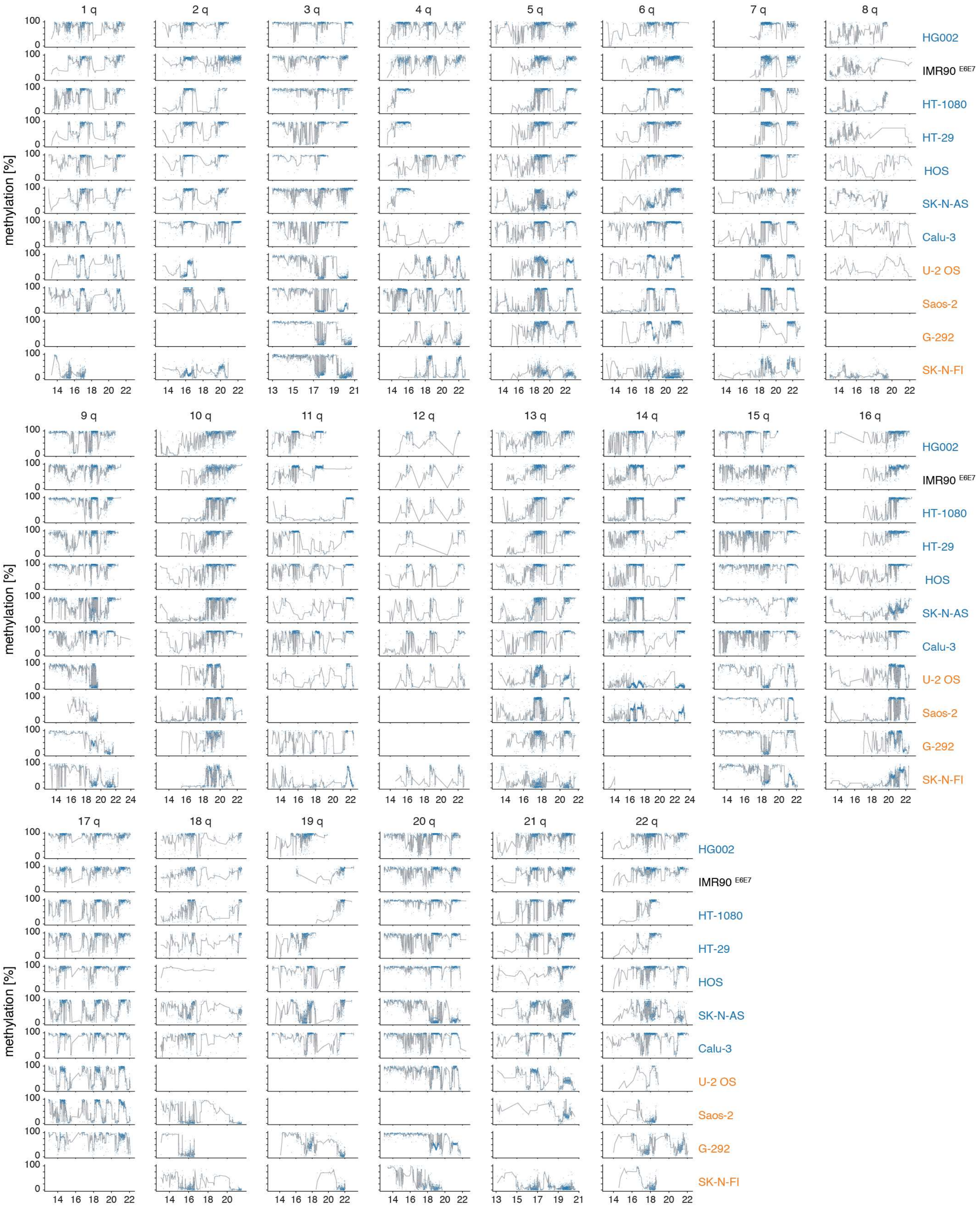

Supplementary Fig. 9. Subtelomeric methylation q chromosome arms. Line graphs of subtelomeric CpG islands closest to telomeres. The percentage of methylation is shown for indicated cell lines and chromosome arms at their kilobase position. Individual CpG sites are represented by blue points and the rolling methylation median is shown as a gray line with a

window size of 4. For IMR90 ^{E6E7} the methylation at population doubling 66.2 is shown. Cell lines are color-coded according to telomere maintenance mechanism (TMM); cells with no TMM are in black, telomerase-positive cells in blue and ALT-positive cells in orange. Only data for chromosomes with at least 10 reads per arm are shown. Data is based on the pooled analysis of one or two experiments per cell line. See Supplementary Tables 1, 2 and 6 for details.

Supplementary Table 1. Telo-seq summary HG002.

	HG002 replicate #1	HG002 replicate #2
**Flow cell**		
Flow cell ID	FAV29018	FAR62417
No. total reads	991785	683252
No. non-telomeric reads	947095	673956
No. telomeric reads	44690	9296
% telomeric reads / total reads	4.51	1.36
% C-strand / telomeric reads	99.19	99.07
No. mapped Q20+ telomeric reads (% telomeric reads)	39808 (89.08)	7930 (85.31)
**Telomere length**		
Mean [bp]	5247	5270
Standard deviation [bp]	2085	2135
Min [bp]	48	51
Lower quartile [bp]	4011	4019
Median [bp]	4979	4990
Upper quartile [bp]	6115	6164
Max [bp]	34478	17890
IQR [bp]	2104	2145
CV	0.40	0.41
% telomeres < 1 kb	1.20	1.39
% telomeres >10 kb	3.20	3.39

Supplementary Table 2. Telo-seq summary IMR90 ^{E6E7} progression.

	IMR90 ^{E6E7}					
Population doublings	66.2	68.7	71.4	85.0	96.0	106.1
**Flow cell**						
Flow cell ID	FAU74032	FAU64354	FAV31378	FAW19571	FAW29975	FAV35454
No. total reads	677351	674509	391804	789868	434826	515593
No. non-telomeric reads	645050	651266	373105	762399	401417	502458
No. telomeric reads	32301	23243	18699	27469	33409	13135
% telomeric reads / total reads	4.77	3.45	4.77	3.48	7.68	2.55
% C-strand / telomeric reads	99.22	98.98	98.99	99.29	99.43	98.39
No. mapped Q20+ telomeric reads (% telomeric reads)	19288 (59.71)	13649 (58.72)	11159 (59.68)	16287 (59.29)	19978 (59.80)	7412 (56.43)
**Telomere length**						
Mean [bp]	4276	4252	4176	3691	3211	2746
Standard deviation [bp]	2610	2606	2597	2381	2219	2161
Min [bp]	48	50	49	50	49	51
Lower quartile [bp]	2359	2340	2246	1932	1561	1221
Median [bp]	3808	3811	3713	3223	2732	2176
Upper quartile [bp]	5668	5650	5599	4945	4325	3587
Max [bp]	26695	35280	28771	27151	27148	27340
IQR [bp]	3309	3310	3353	3013	2764	2366
CV	0.61	0.61	0.62	0.65	0.69	0.79
% telomeres <1 kb	4.98	5.33	5.97	7.78	11.99	18.32
% telomeres >10 kb	3.24	3.08	2.96	1.73	1.13	0.99

Supplementary Table 3. Telo-seq summary fibroblasts of aging cohort.

	fibroblasts							
Sample ID	iPSCORE_2_11	iPSCORE_7_5	3438	3449	3342	3551	27	40
Donor age [years]	20	25	66	73	78	82	86	94
Sex	F	M	F	F	F	F	M	M
**Flow cell**								
Flow cell ID	FAW54670	FAT60593	FAW81431	FAW81279	FAW52127	FAW54411	FAW51887	FAW51781
No. total reads	424071	478605	221582	248262	484072	839184	918184	1031463
No. non-telomeric reads	409936	457502	211360	227344	461460	810353	864266	1012629
No. telomeric reads	14135	21103	10222	20918	22612	28831	53918	18834
% telomeric reads / total reads	3.33	4.41	4.61	8.43	4.67	3.44	5.87	1.83
% C-strand / telomeric reads	99.24	99.12	99.41	99.63	99.44	99.29	99.65	99.01
No. mapped Q20+ telomeric reads (% telomeric reads)	8699 (61.54)	11828 (56.05)	6059 (59.27)	12858 (61.47)	13555 (59.95)	16832 (58.38)	33667 (62.44)	11813 (62.72)
**Telomere length**								
Mean [bp]	7216	7696	6351	5575	5844	7286	5077	5536
Standard deviation [bp]	2862	3359	2780	2407	2619	2870	2386	2885
Min [bp]	51	51	54	52	51	47	51	48
Lower quartile [bp]	5362	5551	4550	3962	4130	5417	3424	3457
Median [bp]	6850	7296	6147	5397	5685	7097	4824	5179
Upper quartile [bp]	8566	9536	7852	6948	7185	8918	6393	7214
Max [bp]	29857	30573	29285	53581	31714	32488	25122	29966
IQR [bp]	3204	3985	3302	2986	3055	3502	2969	3758
CV	0.40	0.44	0.44	0.43	0.45	0.39	0.47	0.52
% telomeres < 1 kb	0.62	0.87	1.48	1.42	0.84	0.77	1.57	2.67
% telomeres >10 kb	13.66	20.54	8.83	3.93	5.17	14.87	3.48	7.78

Supplementary Table 4. Telo-seq summary iPSCs.

	iPSCs					
Sample ID	iPSCORE _7_5	3449	3342	3551	27	40
Donor age [years]	25	73	78	82	86	94
Sex	M	F	F	F	M	M
Flow cell						
Flow cell ID	FAT57861	FAW77335	FAW81441	FAW62301	FAW81293	FAW81430
No. total reads	467581	299571	235974	255072	204477	190395
No. non-telomeric reads	463957	296061	232315	249445	199007	186521
No. telomeric reads	3624	3510	3659	5627	5470	3874
% telomeric reads / total reads	0.78	1.17	1.55	2.21	2.68	2.03
% C-strand / telomeric reads	99.06	98.40	98.52	98.68	99.31	99.15
No. mapped Q20+ telomeric reads (% telomeric reads)	1614 (44.53)	1780 (50.71)	1699 (46.43)	2835 (50.38)	3165 (57.86)	2312 (59.68)
**Telomere length**						
Mean [bp]	8723	9851	10091	10150	8679	8731
Standard deviation [bp]	3998	3550	3260	3045	2693	2754
Min [bp]	49	53	51	50	53	54
Lower quartile [bp]	6392	7765	8340	8440	7043	7084
Median [bp]	9373	10004	10328	10235	8625	8622
Upper quartile [bp]	11499	12128	12137	12047	10308	10314
Max [bp]	25962	26875	30382	30800	22454	27440
IQR [bp]	5107	4363	3797	3608	3265	3230
CV	0.46	0.36	0.32	0.30	0.31	0.32
% telomeres < 1 kb	5.57	1.51	1.45	1.10	0.86	0.96
% telomeres >10 kb	42.72	50.03	54.71	53.67	29.29	28.83

Supplementary Table 5. Telo-seq summary cancer cell lines.

	HT-1080	HT-29	HOS	SK-N-AS	Calu-3	U-2 OS	Saos-2	G-292	SK-N-FI	SK-LU-1
TMM	TERT+	TERT+	TERT+	TERT+	TERT+	ALT+	ALT+	ALT+	ALT+	ALT+
**Flow cell**										
No. flow cells	1	1	1	2	1	2	2	2	1	4
No. total reads	247970	1072547	622756	833191	516610	1433769	907310	1294065	545517	1564687
No. non-telomeric reads	227802	1044035	596826	753245	441546	1383594	844568	1252757	513768	1562819
No. telomeric reads	20168	28512	25930	79946	75064	50175	62742	41308	31749	1868
% telomeric reads / total reads	8.13	2.66	4.16	9.60	14.53	3.50	6.92	3.19	5.82	0.12
% C-strand / telomeric reads	99.70	99.80	99.56	99.35	99.69	99.22	99.52	99.39	99.39	95.87
No. mapped Q10+ telomeric reads (% telomeric reads)	14839 (73.58)	19864 (69.67)	19859 (76.59)	37353 (46.72)	54675 (72.84)	15844 (31.58)	21271 (33.90)	12079 (29.24)	18069 (56.91)	803 (42.99)
**Telomere length**										
Mean [bp]	3357	6040	2755	5233	1522	8096	3956	6193	6750	2197
Standard deviation [bp]	1208	2864	971	2062	817	8694	3272	5570	7448	2648
Min [bp]	50	50	51	47	50	50	49	50	50	49
Lower quartile [bp]	2582	3979	2098	3796	1019	2177	1686	2293	2141	572
Median [bp]	3240	5733	2663	5104	1406	5184	3108	4688	4311	1374
Upper quartile [bp]	3974	7719	3328	6535	1866	10865	5284	8381	8529	2832
Max [bp]	12853	46668	14562	26026	16890	134722	52996	57078	108011	26053
IQR [bp]	1392	3740	1230	2739	847	8688	3598	6088	6388	2260
CV	0.36	0.47	0.35	0.39	0.54	1.07	0.83	0.90	1.10	1.21
% telomeres < 1 kb	1.01	1.23	2.11	1.01	23.84	11.00	12.90	10.39	8.65	39.78
% telomeres >10 kb	0.05	8.93	0.00	1.84	0.01	27.60	5.34	18.33	20.15	2.41

Supplementary Table 6. Telo-seq summary cancer cell lines per flow cell. a, general flow cell information b, telomere length information.

a, Flow cell

Sample	Flow cell ID	No. total reads	No. non-telomeric reads	No. telomeric reads	% telomeric reads / total reads	% C-strand / telomeric reads	No. mapped Q10+ telomeric reads (% telomeric reads)
HT-1080	FAV27627	247970	227802	20168	8.13	99.70	14839 (73.58)
HT-29	FAU93874	1072547	1044035	28512	2.66	99.80	19864 (69.65)
HOS	FAV28723	622756	596826	25930	4.16	99.56	19859 (76.59)
SK-N-AS	FAV35348	454312	395236	59076	13.00	99.32	21916 (37.10)
SK-N-AS	FAW30055	378879	358009	20870	5.51	99.43	15437 (73.97)
Calu-3	FAW31323	516610	441546	75064	14.53	99.69	54675 (72.84)
U-2 OS	FAV89509	578352	563859	14493	2.51	99.10	4534 (31.28)
U-2 OS	FAW19540	855417	819735	35682	4.17	99.27	11310 (31.67)
Saos-2	FAV92737	448271	440182	8089	1.80	99.04	2783 (34.40)
Saos-2	FAW30039	459039	404386	54653	11.91	99.59	18488 (33.83)
G-292	FAT60863	983283	968662	14621	1.49	99.10	4264 (29.16)
G-292	FAW29984	310782	284095	26687	8.59	99.55	7815 (29.28)
SK-N-F1	FAV34018	545517	513768	31749	5.82	99.39	18069 (56.91)
SK-LU-1	FAU08605	428519	428182	337	0.08	95.25	131 (38.88)
SK-LU-1	FAV21836	321923	321549	374	0.12	93.85	154 (41.18)
SK-LU-1	FAV35378	420501	419950	551	0.13	96.91	226 (41.01)
SK-LU-1	FAW30048	393744	393138	606	0.15	96.53	292 (48.18)

b, Telomere length

Sample	Flow cell ID	Mean [bp]	Standard deviation [bp]	Min [bp]	Lower quartile [bp]	Median [bp]	Upper quartile [bp]	Max [bp]	IQR [bp]	CV	% telomeres < 1 kb	% telomeres >10 kb
HT-1080	FAV27627	3357	1208	50	2582	3240	3974	12853	1392	0.36	1.01	0.05
HT-29	FAU93874	6040	2864	50	3979	5733	7719	46668	3740	0.47	1.23	8.93
HOS	FAV28723	2755	971	51	2098	2663	3328	14562	1230	0.35	2.11	0.00
SK-N-AS	FAV35348	5246	2061	50	3805	5118	6548	20460	2743	0.39	0.94	1.88
SK-N-AS	FAW30055	5198	2064	47	3766	5074	6493	26026	2727	0.40	1.19	1.73
Calu-3	FAW31323	1522	817	50	1019	1406	1866	16890	847	0.54	23.84	0.01
U-2 OS	FAV89509	7090	7282	51	2009	4722	9606	134722	7597	1.03	11.93	23.78
U-2 OS	FAW19540	8505	9175	50	2253	5395	11466	104101	9213	1.08	10.62	29.15
Saos-2	FAV92737	3945	3296	51	1673	3101	5268	46266	3595	0.84	13.40	5.44
Saos-2	FAW30039	3958	3268	49	1689	3109	5286	52996	3597	0.83	12.83	5.33
G-292	FAT60863	6202	5545	50	2320	4720	8351	56378	6031	0.89	10.23	18.28
G-292	FAW29984	6189	5583	50	2278	4669	8393	57078	6115	0.90	10.48	18.35
SK-N-F1	FAV34018	6750	7448	50	2141	4311	8529	108011	6388	1.10	8.65	20.15
SK-LU-1	FAU08605	2072	2555	50	556	1333	2619	23465	2063	1.23	41.54	1.78
SK-LU-1	FAV21836	2035	2442	52	490	1218	2796	20631	2307	1.20	43.32	1.87
SK-LU-1	FAV35378	2268	2564	51	595	1435	3055	20515	2460	1.13	38.48	2.54
SK-LU-1	FAW30048	2302	2884	49	619	1434	2800	26053	2181	1.25	37.79	2.97

Supplementary Table 7. Telo-seq oligos.

Name	Sequence 5’ → 3’
T1	/5Phos/AGCAATACGTAACTGAACGAAGTCCCTAACCCTAACCCTAA
T2	/5Phos/AGCAATACGTAACTGAACGAAGTTAACCCTAACCCTAACCC
T3	/5Phos/AGCAATACGTAACTGAACGAAGTCTAACCCTAACCCTAACC
T3	/5Phos/AGCAATACGTAACTGAACGAAGTCCTAACCCTAACCCTAAC
T5	/5Phos/AGCAATACGTAACTGAACGAAGTAACCCTAACCCTAACCCT
T6	/5Phos/AGCAATACGTAACTGAACGAAGTACCCTAACCCTAACCCTA
S1	ACTTCGTTCAGTTACGTATTGCTAGCAAT
