# Supplementary material for: High resolution long-read telomere sequencing reveals dynamic mechanisms in aging and cancer
Source: Nat Commun. 2024 Jun 18;15:5149. doi: 10.1038/s41467-024-48917-7 (PMC11189484; doi:10.1038/s41467-024-48917-7)
Supplement: Supplementary file 3 — Description of Additional Supplementary Files [file 41467_2024_48917_MOESM3_ESM.pdf]

Description of Additional Supplementary Files

File Name: Supplementary Data 1
Description: Telo-seq summary HG002 chromosome arm-specific telomere

File Name: Supplementary Data 2
Description: Telo-seq summary IMR90 ^{E6E7} progression chromosome-arm specific telomere length.

File Name: Supplementary Data 3
Description: Telo-seq summary HG002 allele-specific telomere length.

File Name: Supplementary Data 4
Description: Telo-seq summary fibroblasts of aging cohort chromosome-arm specific telomere length.

File Name: Supplementary Data 5
Description: Telo-seq summary iPSCs chromosome-arm specific telomere length.
