# Supplementary material for: High resolution long-read telomere sequencing reveals dynamic mechanisms in aging and cancer
Source: Nat Commun. 2024 Jun 18;15:5149. doi: 10.1038/s41467-024-48917-7 (PMC11189484; doi:10.1038/s41467-024-48917-7)
Supplement: Supplementary file 9 — Reporting Summary [file 41467_2024_48917_MOESM9_ESM.pdf]

nature portfolio

Corresponding author(s): Scott Hickey and Jan Karlseder
NCOMMS-23-51397-T

Last updated by author(s): Apr 30, 2024

Reporting Summary

Nature Portfolio wishes to improve the reproducibility of the work that we publish. This form provides structure for consistency and transparency in reporting. For further information on Nature Portfolio policies, see our Editorial Policies and the Editorial Policy Checklist.

Statistics

For all statistical analyses, confirm that the following items are present in the figure legend, table legend, main text, or Methods section.

n/a	Confirmed	
☐	☒	The exact sample size (*n*) for each experimental group/condition, given as a discrete number and unit of measurement
☐	☒	A statement on whether measurements were taken from distinct samples or whether the same sample was measured repeatedly
☐	☒	The statistical test(s) used AND whether they are one- or two-sided<br>*Only common tests should be described solely by name; describe more complex techniques in the Methods section.*
☒	☐	A description of all covariates tested
☒	☐	A description of any assumptions or corrections, such as tests of normality and adjustment for multiple comparisons
☐	☒	A full description of the statistical parameters including central tendency (e.g. means) or other basic estimates (e.g. regression coefficient) AND variation (e.g. standard deviation) or associated estimates of uncertainty (e.g. confidence intervals)
☐	☒	For null hypothesis testing, the test statistic (e.g. *F*, *t*, *r*) with confidence intervals, effect sizes, degrees of freedom and *P* value noted<br>*Give P values as exact values whenever suitable.*
☒	☐	For Bayesian analysis, information on the choice of priors and Markov chain Monte Carlo settings
☒	☐	For hierarchical and complex designs, identification of the appropriate level for tests and full reporting of outcomes
☒	☐	Estimates of effect sizes (e.g. Cohen's *d*, Pearson's *r*), indicating how they were calculated

Our web collection on statistics for biologists contains articles on many of the points above.

Software and code

Policy information about availability of computer code

Data collection	All software used has been indicated in the methods section.<br>Code and software submission-please see software checklist and below.<br>Telo-seq analysis pipeline is available on https://github.com/priyesh000/teloseq [https://doi.org/10.5281/zenodo.11081583]
Data analysis	GraphPad Prism (version 8.4.3), R (version 4.3.1), Python (version 3.8.10) , TeloTool (version 1.3), WALTER (version 2.0), Bonito (version 0.6.2) , Noise Cancelling Repeat Finder algorithm (version 1.01.00 20190426) , Minimap2 (version 2.22), Dorado (version 0.3.4, dna_r9.4.1_e8_sup@v3.3_5mC_5hmC model), and Modkit (version0.11.1)<br>See methods for details.

For manuscripts utilizing custom algorithms or software that are central to the research but not yet described in published literature, software must be made available to editors and reviewers. We strongly encourage code deposition in a community repository (e.g. GitHub). See the Nature Portfolio guidelines for submitting code & software for further information.

Data

Policy information about availability of data

All manuscripts must include a data availability statement. This statement should provide the following information, where applicable:

- Accession codes, unique identifiers, or web links for publicly available datasets
- A description of any restrictions on data availability
- For clinical datasets or third party data, please ensure that the statement adheres to our policy

All data, code, and materials used in the analysis are archived at the Salk Institute and Oxford Nanopore Technologies.
Raw sequencing data is deposited at https://www.ncbi.nlm.nih.gov/bioproject/PRJNA1040425.
Source data is provided with the manuscript as a Source Data file.
Software is deposited at https://github.com/priyesh000/teloseq [https://doi.org/10.5281/zenodo.11081583].

Research involving human participants, their data, or biological material

Policy information about studies with human participants or human data. See also policy information about sex, gender (identity/presentation), and sexual orientation and race, ethnicity and racism.

Reporting on sex and gender	Sex information is provided for donor-derived human cell samples.
Reporting on race, ethnicity, or other socially relevant groupings	This information is not available for human cell samples due to blinding.
Population characteristics	Only the age and sex are available for human cell samples.
Recruitment	ADRC (Alzheimer's Disease Research Center) participants at UCSD have given broad consent to a range of experiments, including skin fibroblast and induced pluripotent stem cell derivation, cell engineering, and genetic sequencing and manipulation prior to providing a skin biopsy. The consent form is available upon request.
Ethics oversight	See above.

Note that full information on the approval of the study protocol must also be provided in the manuscript.

Field-specific reporting

Please select the one below that is the best fit for your research. If you are not sure, read the appropriate sections before making your selection.

☒ Life sciences ☐ Behavioural & social sciences ☐ Ecological, evolutionary & environmental sciences

For a reference copy of the document with all sections, see nature.com/documents/nr-reporting-summary-flat.pdf

Life sciences study design

All studies must disclose on these points even when the disclosure is negative.

Sample size	This is an exploratory study, not a comparative one.
Data exclusions	No data were excluded.
Replication	Replication and reproducibility indicted in Tables S1 and S6.
Randomization	No randomization was made.
Blinding	Cell line names were blinded prior to sequencing at ONT.

Reporting for specific materials, systems and methods

We require information from authors about some types of materials, experimental systems and methods used in many studies. Here, indicate whether each material, system or method listed is relevant to your study. If you are not sure if a list item applies to your research, read the appropriate section before selecting a response.

Materials & experimental systems

n/a	Involved in the study
☐	☒ Antibodies
☐	☒ Eukaryotic cell lines
☒	☐ Palaeontology and archaeology
☒	☐ Animals and other organisms
☒	☐ Clinical data
☒	☐ Dual use research of concern
☒	☐ Plants

Methods

n/a	Involved in the study
☒	☐ ChIP-seq
☒	☐ Flow cytometry
☒	☐ MRI-based neuroimaging

Antibodies

Antibodies used: anti-digoxigenin-AP, Fab fragments (Roche, 11093274910, 1:2000 dilution in blocking solution)

Validation: Anti-digoxigenin-AP, Fab fragments for TRF have been in characterized in Kimura M et al., 2015 (PMID 21085125) and Lai T et al., 2016 (PMID: 27286808).
Information provided by the Manufacturer's website (https://www.sigmaaldrich.com/US/en/product/roche/11093274910#product-documentation) include:
- Cross reactivity to digitoxin and digitoxigenin: <1 %
- No cross reactivity with other human estrogen or androgen steroids, e.g. estradiol or testosterone
- Cross reactivity with digoxin: not known
- Conjugate does not bind to itself at all
- Normally one molecule of the conjugate binds to one molecule digoxigenin, although there are two possible binding sites for digoxigenin

Eukaryotic cell lines

Policy information about cell lines and Sex and Gender in Research

Cell line source(s): Calu-3 (HTB-55), G-292, clone A141B1 (CRL-1423), HOS (CRL-1543), HT-1080 (CCL-121), HT-29 (HTB-38), Saos-2 (HTB85), SK-LU-1 (HTB-57), SK-N-AS (CRL-2137), SK-N-FI (CRL-2142) and U-2 OS (HTB96) cancer cell lines and IMR90 (CCL-186) fibroblasts were purchased from ATCC.
The lymphoblastoid cell line HG002 (GM24385) was purchased from Coriell Institute for Medical Research.
Donor-derived fibroblasts were collected at University of California, San Diego (UCSD) and are part of the Salk AHA-Allen aging cohort.Alzheimer’s Disease Research Center participants at UCSD have given broad consent to a range of experiments, including skin fibroblast and induced pluripotent stem cell derivation, cell engineering, and genetic sequencing and manipulation prior to providing a skin biopsy. Sex of donor-derived fibroblast is: iPSCORE_2_11 female; iPSCORE_7_5 male; 3438 female; 3449 female; 3342 female; 3551 female; 27 male; 40 male.

Authentication: Outlined in methods.

Mycoplasma contamination: All cell lines are routinely tested and only used if negative.

Commonly misidentified lines (See ICLAC register): All lines used have been authenticated by the commercial providers and no commonly misidentified cell lines have been used.

Plants

Seed stocks: No plants have been used.

Novel plant genotypes: *Describe the methods by which all novel plant genotypes were produced. This includes those generated by transgenic approaches, gene editing, chemical/radiation-based mutagenesis and hybridization. For transgenic lines, describe the transformation method, the number of independent lines analyzed and the generation upon which experiments were performed. For gene-edited lines, describe the editor used, the endogenous sequence targeted for editing, the targeting guide RNA sequence (if applicable) and how the editor was applied.*

Authentication: *Describe any authentication procedures for each seed stock used or novel genotype generated. Describe any experiments used to assess the effect of a mutation and, where applicable, how potential secondary effects (e.g. second site T-DNA insertions, mosiacism, off-target gene editing) were examined.*
